# Complete cysteine-scanning mutagenesis of the *Salmonella typhimurium* melibiose permease

**DOI:** 10.1016/j.jbc.2021.101090

**Published:** 2021-08-18

**Authors:** Kelsey J. Markham, Elena B. Tikhonova, Aaron C. Scarpa, Parameswaran Hariharan, Satoshi Katsube, Lan Guan

**Affiliations:** Department of Cell Physiology & Molecular Biophysics, Center for Membrane Protein Research, School of Medicine, Texas Tech University Health Sciences Center, School of Medicine, Lubbock, Texas, USA

**Keywords:** MelB_St_, cation-coupled symporter, structure and function analysis, Cys-scanning mutagenesis, MFS transporter, α-NPG, α-nitrophenyl galactoside, Cys-less, 4 endogenous Cys was replaced by Ala, Cys-scanning mutagenesis, Cysteine-scanning mutagenesis, DDMB, dodecyl melibioside, DW2 strain, *mel*A^+^, Δ*melB*, Δ*lacZY*, MelB_Ec_, the melibiose permease of *E. coli*, MelB_St_, the melibiose permease of *Salmonella typhimurium*, MFS, major facilitator superfamily

## Abstract

The melibiose permease of *Salmonella typhimurium* (MelB_St_) catalyzes the stoichiometric symport of galactopyranoside with a cation (H^+^, Li^+^, or Na^+^) and is a prototype for Na^+^-coupled major facilitator superfamily (MFS) transporters presenting from bacteria to mammals. X-ray crystal structures of MelB_St_ have revealed the molecular recognition mechanism for sugar binding; however, understanding of the cation site and symport mechanism is still vague. To further investigate the transport mechanism and conformational dynamics of MelB_St_, we generated a complete single-Cys library containing 476 unique mutants by placing a Cys at each position on a functional Cys-less background. Surprisingly, 105 mutants (22%) exhibit poor transport activities (<15% of Cys-less transport), although the expression levels of most mutants were comparable to that of the control. The affected positions are distributed throughout the protein. Helices I and X and transmembrane residues Asp and Tyr are most affected by cysteine replacement, while helix IX, the cytoplasmic middle-loop, and C-terminal tail are least affected. Single-Cys replacements at the major sugar-binding positions (K18, D19, D124, W128, R149, and W342) or at positions important for cation binding (D55, N58, D59, and T121) abolished the Na^+^-coupled active transport, as expected. We mapped 50 loss-of-function mutants outside of these substrate-binding sites that suffered from defects in protein expression/stability or conformational dynamics. This complete Cys-scanning mutagenesis study indicates that MelB_St_ is highly susceptible to single-Cys mutations, and this library will be a useful tool for further structural and functional studies to gain insights into the cation-coupled symport mechanism for Na^+^-coupled MFS transporters.

Secondary active transporters play important roles in human health and disease. Melibiose permease of *Salmonella typhimurium* (MelB_St_) is a secondary active transporter catalyzing stoichiometric cotransport of galactopyranoside with a cation (either H^+^, Na^+^, or Li^+^). MelB belongs to the glycoside-pentoside-hexuronide:cation symporter family in the Transporter Classification Database (TCDB) (1), which is a subgroup of the major facilitator superfamily (MFS) of membrane transporters found from bacteria to mammals. MelB homologues in human play important roles from lipid uptake (such as MFSD2A) (2) to cancer development (MFSD13A) (3, 4). Based on the larger Pfam database, these proteins belong to MFS_2 family (5) with greater than 11,000 sequenced genes across thousands of species. The first high-resolution structure of a MFS_2 family transporter was obtained from MelB_St_ (6). More recently, the substrate-bound crystal structure of MelB_St_ (7) and a cryo-EM structure of MFSD2A (8) have been determined.

MelB as an early discovered transporter poses several advantages as a model system for understanding the secondary active transport, especially for Na^+^-coupled symport. It is well expressed in *Escherichia coli* and purified protein is stable. MelB_St_ and the *E. coli* MelB (MelB_Ec_) have been subjected to varied biochemical and biophysical studies including X-ray crystallographic analyses, as well as the development of novel analytical techniques for membrane protein research (6, 7, 9, 10, 11, 12, 13, 14, 15, 16, 17, 18, 19, 20, 21, 22, 23). The 3D crystal structures of MelB_St_ confirmed the previous computational predictions (19, 24). MelB adopts a typical MFS fold, composed of 12 transmembrane α-helices organized into two pseudo-symmetric bundles (Fig. 1). An internal cavity is formed by eight transmembrane helices (I, II, IV, V, VII, VIII, X, and XI), and one molecule galactoside is bound at the apex of the outward-facing cavity. The rest four transmembrane helices (III, VI, IX, XII) lie on the periphery of the molecule and interact with membrane lipids.

**Figure 1:**
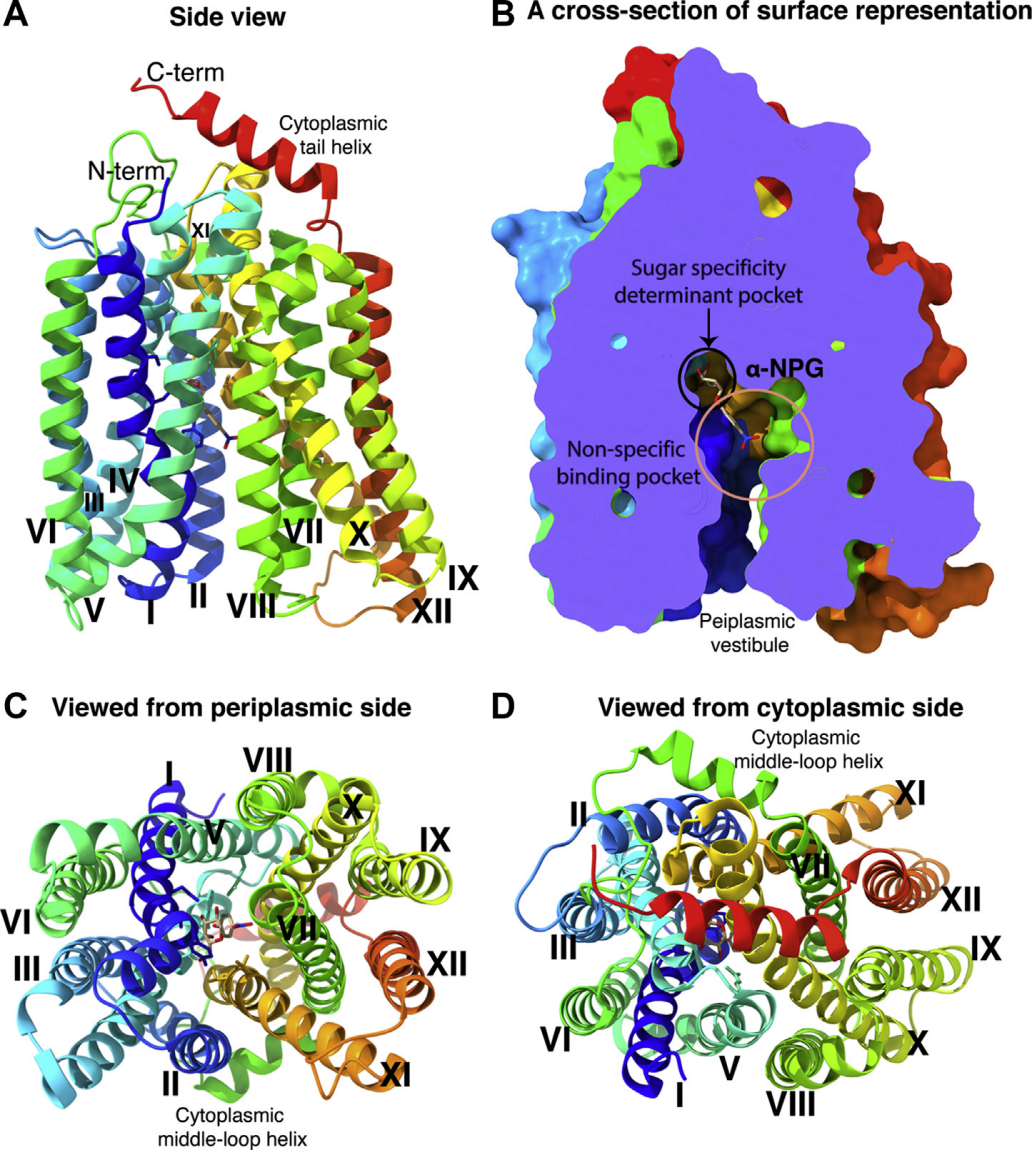
**Ligand-bound outward-facing crystal structure of D59C MelB**_**St**_**.***A*, overall fold and helical packing [PDB ID, 7L17]. The D59C MelBSt structure bound with α-NPG is viewed parallel to the membrane and shown in *cartoon* representation colored in rainbow from N-terminus in *blue* to C-terminal end in *red*. The cytoplasmic tail helix is indicated. Transmembrane helix is numbered in *Roman numerals*. *B*, a cross section of surface representation with the N-terminal bundle orientated at the *left side*. The sugar specificity determinant pocket and nonspecific binding pocket are indicated by *circles*. *C* and *D*, viewed from periplasmic (*C*) and cytoplasmic sides (*D*) of MelB_St_ at a sugar-bound outward-facing conformation, respectively.

The current new sugar-bound crystal structures with α-nitrophenyl galactoside (α-NPG) or dodecyl melibioside (DDMB) were determined from a MelB_St_ uniporter mutant D59C with a Cys mutation at the highly conserved cation-binding position Asp59 (7). This D59C mutation completely abolishes the cation binding and all three modes of cation-coupled melibiose active transport. Interestingly, it ferments melibiose on the MacConkey agar plates containing melibiose as the sole carbohydrate source (7) and catalyzes the melibiose exchange in membrane vesicles at a rate similar to that of the wild type (6). In the sugar-binding site, the binding of galactosyl moiety on both ligands with MelB_St_ is essentially same, so the sugar-specificity determinant pocket in MelB is clearly defined (7). Briefly, both N- and C-terminal bundles including five helices (I, IV, V, X, and XI), particularly helices I and IV, are engaged in determining the sugar specificity. Asp19 (helix I) and Asp124 (IV), the two major hydrogen-bond (H-bond) acceptors, as well as Lys18 (I), Trp128 (IV), and Arg149 (V), the major H-bond donors, are around the galactosyl moiety. Trp342 provides a π-carbon interaction with the galactosyl ring and forms aromatic stacking with the phenyl ring or the glucosyl ring.

Regarding the cation site, two negatively charged Asp55 and Asp59 on helix II of MelB_Ec_ have been identified to be the Na^+^-binding residues (19, 25, 26). Asp55 forms a salt-bridge interaction with Lys377 on helix XI, and position 59 is at a close distance to the C6-OH group of the bound sugar (7). Systematic thermodynamics analyses of ligand binding to MelB_St_ have shown that a positive cooperativity exists between melibiose and its coupling cations, at a cooperativity number of 8 with Na^+^ and 2 with H^+^ (21, 27); *i.e.*, binding to any binary state has higher affinity than that to the apo MelB_St_. The observed close distance between the position Asp59 and the C6-OH on the specific galactosyl ring provided a structural basis for the cooperative binding mechanism. While the structural details are still unclear, the cooperative binding of the two coupled substrates is the core mechanism of symport by ensuring obligatory coupling and overcoming energetic barriers during transport (21, 23, 27).

MelB has been proposed to utilize alternating-access process for transport; *i.e.*, MelB exposes its substrate-binding sites to either cytoplasmic or periplasmic surfaces of the transporter in an alternating manner to complete the transport cycle. Although only the outward-facing conformation is resolved, MelB should carry the cosubstrate from the outward-facing state to inward-facing state through occluded intermediates, as demonstrated in other MFS transporters (8, 28, 29, 30, 31). There are still many unresolved questions about the cation site, coupling mechanism, conformational changes, and symporter mechanism. The high-resolution structure only represents static postures but misses the conformational dynamics information required for our understanding of transport mechanisms. A simple but systematic approach combined with available high-resolution 3-D structural data can provide more information for further mechanistic studies. Therefore, a complete Cys-scanning mutagenesis was applied to identify key positions for creating a functional map or framework that will help address some of the dynamics of the transporter through further biochemical and biophysical analysis. In LacY, Cys-scanning mutagenesis combined with chemical modifications and site-directed mutagenesis were first successfully applied, which provided a meaningful structure–function relationship, determined critical positions for ligand binding, and precise labeling sites for dynamic studies (29, 32, 33). A fully functional Cys-less MelB_St_ mutant was generated with all four endogenous Cys residues replaced by residue Ala; then placement of Cys at each position, one at a time, was carried out, which yielded a 476 single-Cys mutant library covering the full-length MelB_St_. The entire library was analyzed with melibiose transport and fermentation assay; for affected positions, the protein expression levels were determined by western blotting. The data show that the positions critical for melibiose active transport are widely distributed throughout the protein. While many of them are frequently mapped in sugar-binding site or cation-binding pocket, as well as the cytoplasmic salt-bridge network, a large number of unique mutants are located far from these places, some of which have not been specifically studied.

## Results

### Functional characterization of 476 single-Cys mutants

*E. coli* DW2 (*melA*^+^Δ*melB*Δ*lacZY*) intact cells expressed the Cys-less MelB_St_ or individual single-Cys mutants were used to measure Na^+^-coupled melibiose transport activities with a radio-tracer flux technique. For the large number of mutants, [^3^H]melibiose transport at 0.4 mM (10 mCi/mmol) was measured at three time points: zero, 10 s for the determination of the initial uptake and 5 min for a longer accumulation, respectively. The transport activities for each of the 476 mutants are presented by histogram plots (Fig. S1, *A*–*D*). Briefly, the data showed that the function of MelB_St_ is sensitive to single-Cys mutations. The first position as a starting codon was excluded from the data analysis. Among 475 mutants, 226 positions (47.58%) showed initial rates greater than 70% of Cys-less MelB_St_, which are well tolerated to Cys mutation; 218 mutants (45.89%) also showed the accumulation greater than 70% of the control. Helix I contains more transport sensitive positions than other helices; out of all 34 positions on this helix, there are 19 individual single-Cys mutants with initial rate and 5-min accumulation <30% (13 mutants with <15%) of the control; only five mutants have >70% transport activities. On other hand, the cytoplasmic middle-loop, cytoplasmic C-terminal tail, and helix IX contain the least number of positions that is sensitive to Cys mutation.

Melibiose transport activities of all mutants were also studied by melibiose fermentation assay, where cells grown on MacConkey agar plates containing melibiose (at 30 mM) as the sole carbohydrate source. The degree of colony color change to magenta reflects the rate of melibiose transport. The outcome of melibiose fermentation for the 476 mutants was classified into four groups (Fig. S1, *A*–*D*): high fermentation in magenta color (386 mutants), reduced fermentation in pink color (18 mutants), poor fermentation in brown color (seven mutants), and no fermentation in yellow color (65 mutants).

**Figure 2:**
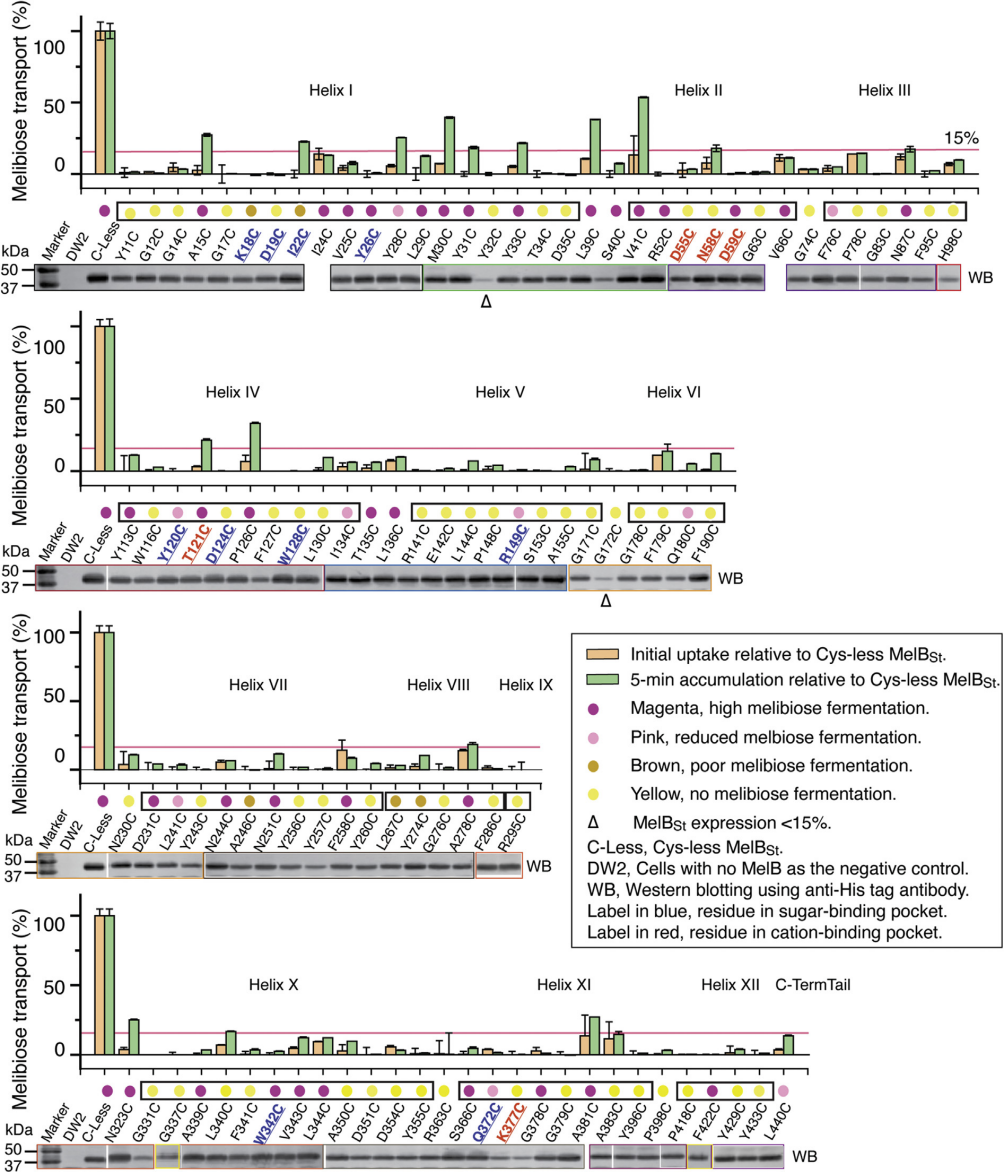
**105 single-Cys mutants with <15% of initial uptake of Cys-less MelB**_**St**_**.** With *E. coli* DW2 strain (*melA*^*+*^*B*^*−*^*lacZ*^*−*^*Y*^*−*^) containing a MelB_St_ mutant, [^3^H]melibiose transport was measured at 0, 10 s, and 5 min at 0.4 mM (at a specific activity of 10 mCi/mmol) in the presence of 20 mM NaCl as described in Experimental procedures. In total, 105 mutants with initial rate <15% were selected and the mean values were presented as histogram bar (*pink bar*, initial uptake; *green bar*, 5-min accumulation). Error bar, standard error from 2 to 3 tests. Activity was normalized by a mean value of Cys-less transport from each batch of tests after corrected by zero point and negative control. Some mutants with less values than the control were presented as zero and the unmodified dataset is presented in Table S1, and unnormalized data are presented in Fig. S1, *A*–*D*. Melibiose fermentation data and protein expression were performed as described in Experimental procedures and placed under transport result. Four colors were used to describe fermentation results as shown in the legend. *Box* covering the fermentation data, transmembrane region as labeled; *pink line*, 15% activity cutoff. WB, Western blots using anti-His tag antibody. Each blot was marked in a *colored box* and their positive (Cys-less MelB_St_) and negative controls (DW2 cell with no MelB) were removed for this presentation. No western blotting data for I24C and V66C mutants, and the blots for G337C and F422C mutants were tested separately.

The results from both melibiose transport and fermentation were further analyzed for these affected positions. A total number of 105 mutants (22.11%) lost melibiose active transport, with initial rate <15% of the control (Fig. 2 and Table S1); most of the mutants have melibiose accumulation <15% of the control, except for 13 mutants with only slightly higher values. Among all 105 mutants with poor transport activities (Table 1), only 57 mutants (54.29%) showed no melibiose fermentation grown as yellow colonies; 34 (32.38%) mutants maintained the high melibiose fermentation although their active transport activity was lost. Remaining nine mutants showed medium (pink) and five low fermentations (brown).

**Table 1:** Distribution of 105 mutants

MelB_St_	Number of mutants with <15% initial uptake	Melibiose fermentation			
		Yellow	Brown	Pink	Magenta
Transmembrane helix	93	51	5	8	29
Extramembrane region	11	6			5
Cytoplasmic middle loop	0				
C-terminal tail	1			1	
Total	105	57 (54.29%)	5 (4.76%)	9 (8.57%)	34 (32.38%)

Yellow, no fermentation; brown, poor fermentation; pink, reduced fermentation; magenta, high fermentation.

The 105 positions are distributed throughout the protein (Fig. 2 and Table 1), 93 mutants located on 12 transmembrane helices including 1 on helix IX, 11 in the extramembrane loops, and 1 at the C-terminal tail. The four inner-lining helices (I, IV, VII, X) have 51 affected positions, accounting for nearly half of mutants with poor transport activities. No mutant within the 105 list was mapped in the 29 residue-long cytoplasmic middle-loop (positions 202–229) or in the last 35 positions on the C-terminal tail.

All 20 amino-acid residues are present in this protein at varied frequencies. The most frequently appeared residues are these hydrophobic residues [Leu (12.0%), Val (9.5%), Ala (9.3%), and Ile (8.4%)] for this hydrophobic membrane protein. Tyr and Asp appear at a frequency of 5.3% and 3.8%, respectively, but these two types of residues are affected by a Cys mutation most frequently (Table 2). Among 25 Tyr residues, 20 positions are in transmembrane helices (80%); at an overall frequency of 68% (17/25) or specific frequency 80% (16/20) in helices (Table 2 and Fig. 2), respectively, Cys replacement, especially on these transmembrane located Tyr, inactivates MelB_St_ transport. Most of the affected Tyr positions are clustered on the helices I and VII. The use of Asp residue in MelB_St_ is less than 5%; among the total of 18 Asp residues, all eight Asp in the transmembrane helices are presented in this 105-mutant list, whereas all other ten Asp residues located in extramembrane regions can tolerate a single-Cys mutation well. Other frequently affected residues including transmembrane Gly, Arg, and Trp are also listed in Table 2.

**Table 2:** Frequency of residues with less than 15% initial uptake (105-list)

MelB_St_	Tyr	Asp	Gly	Glu	Arg	Lys	Trp
Total number in MelB_St_	25	18	36	7	17	14	8
Total number in transmembrane helix	20	8	26	4	10	7	7
Frequency in transmembrane helix (%)	80.00	44.44	72.22	57.14	58.82	50.00	87.5
Number of residues in the 105-list	17	8	14	1	5	2	3
Transmembrane helix							
N-term	8 (9^a^)	5 (5)	7 (14)	(2)	3 (6)	1 (4)	2 (5)
C-term	8 (11)	3 (3)	5 (12)	1 (2)	1 (4)	1 (3)	1 (2)
Extramembrane region							
N-term		(2)	2 (4)	(1)	1 (2)	(1)	(1)
C-term	1 (2)	(3)	(2)		(1)		
Cytoplasmic middle loop	(2)	(1)	(3)	(1)	(1)	(2)	
C-terminal tail	(1)	(4)	(1)	(1)	(3)	(4)	
Affected frequency in full-length MelB_St_ (%)	68.00	44.44	38.89	14.28	29.41	14.28	37.50
Affected frequency in transmembrane helix (%)	80.00	100	46.15	25.00	40	28.57	42.86

Term, terminal bundle.

a The total number present in the defined region.

### Effect of single-Cys mutations on MelB_St_ protein expression

MelB_St_ protein expressions were analyzed with whole cell lysate by western blotting using anti-His tag antibody for 103 mutants with <15% initial melibiose uptake (Fig. 2) and also expressed as the percentage of the Cys-less mutant expression level (Table S1). Interestingly, majority of mutants expressed significant amounts of MelB_St_, and only two mutants (Y32C and G172C) showing less than 15% protein expression of the Cys-less mutant. Tyr32 and Gly172 are located at the periplasmic side of the N-terminal bundle (Fig. S2), and none of them shows melibiose transport or melibiose fermentation. There are few other positions also showing significantly reduced levels in protein expression, including S40C (loop_1–2_), H98C (III), G331C (X), and G378C (XI).

### Single-Cys mutations at the sugar-binding site of MelB_St_

**Figure 3:**
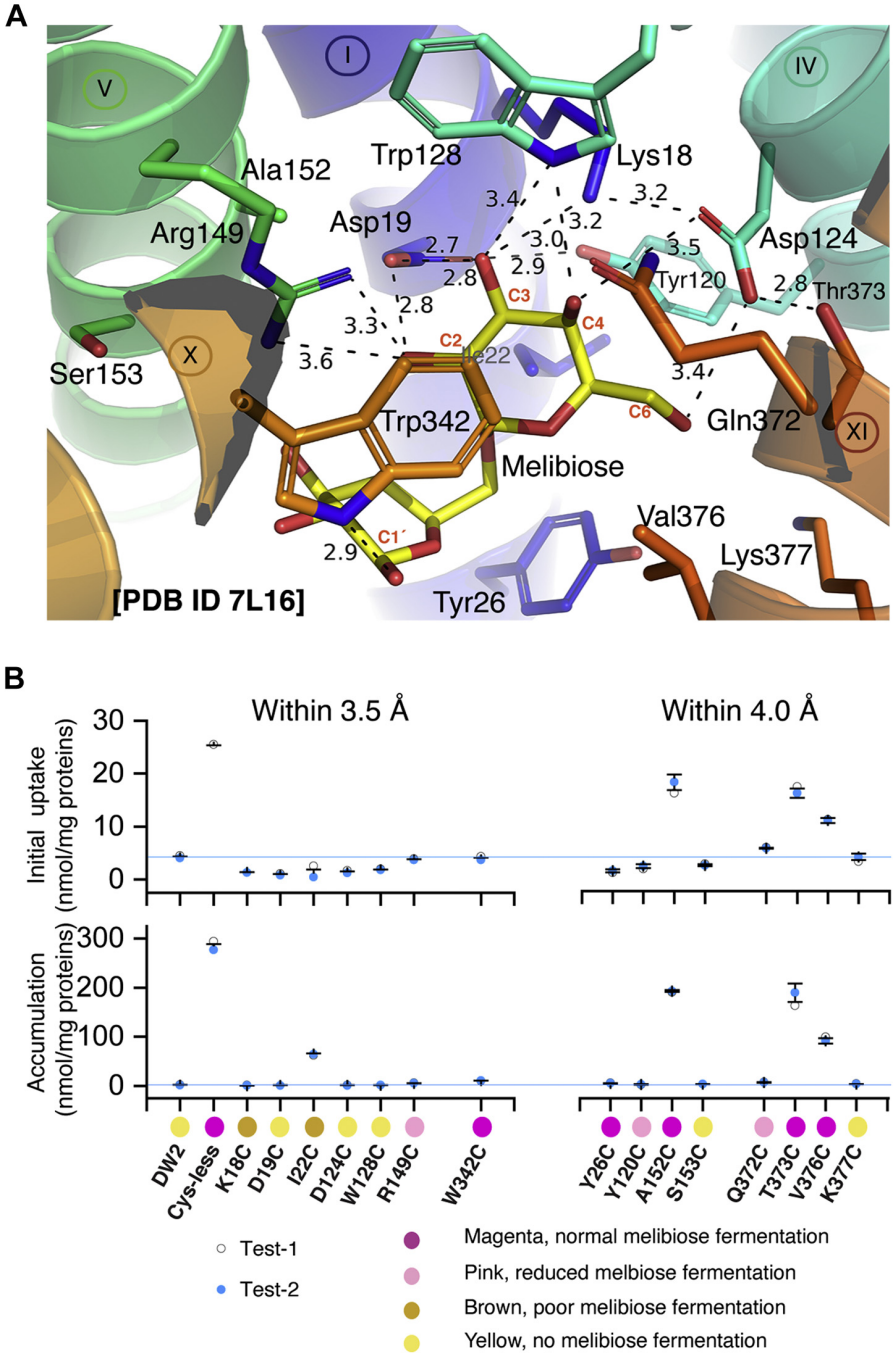
**Mutants in the sugar-binding site.***A*, Cys mutants in the galactoside-binding site. The crystal structure with bound DDMB was used to analyze melibiose-contacting residues after removing the C12 tail [PDB ID, 7L16]. Helices are shown in a *cartoon* representation and labeled in *Roman numerals*; protein sidechains involving in sugar binding are shown in *sticks*, labeled in three letters. Carbon positions on the galactosyl ring are labeled in *red*. H-bond and salt-bridge interactions are judged by distance <3.5 Å and indicated by *dashed lines*. Due to the limited structure resolution, the distance measurement was just used to estimate the relative proximity. *B*, melibiose transport and fermentation. [^3^H]Melibiose transport data and melibiose fermentation results were from Figure 2. *Left* or *right panel*, a collection of residues within a distance of 3.5 Å or 4.0 Å to melibiose molecule. *Magenta circle*, high melibiose fermentation; *pink*, reduced fermentation; *yellow* and *brown circle*, no or poor melibiose fermentation, respectively.

The MelB_St_ structure with bound melibiose-based detergent DDMB [PDB ID, 7L16] was used for the structure and function analysis; the binding site of melibiose was inferred by assuming that the lipid tail of DDMB has negligible effect on the positioning of the carbohydrate moiety. The side chains within 3.5 Å or 4.0 Å distance to the melibiosyl moiety were shown in sticks (Fig. 3*A*). There are seven residues—four charged residue (Lys18, Asp19, Asp124, and Arg149), two Trp (Trp128 and Trp342), and one hydrophobic Ile—are located within 3.5 Å distance to the sugar. Except for the I22C mutant, which showed low level of accumulation at 22% of the control, all other single-Cys mutants completely lost the Na^+^-coupled melibiose transport activity (Fig. 3*B*). There are eight positions at a distance between 3.5 and 4 Å to the sugar, including one positively charged, five polar, and two hydrophobic residues. A Cys replacement at two hydrophobic (Ala152 and Val376) and one polar (Thr373) position showed 40 to 60% of the control with regard to both transport initial and accumulation. All others (including Tyr26, Tyr120, Ser153, Asn372, and Lys377) were inactivated by a Cys mutation. The western blotting analysis showed that these mutants were expressed at a level comparable to the Cys-less mutant; thus, the observed inhibitions on these positions, especially those forming H-bonds with the sugar hydroxyl groups (Lys18, Asp19, Asp124, Trp128, Arg149, and Trp342), likely resulted from loss of sugar-binding affinity, as concluded from those single-site Cys mutants at the same positions in a WT background (6). Notably, not all single-Cys mutations out of the seven binding residues completely lost transport activity. R149C mutant grew on melibiose-containing MacConkey agar plate with pink color indicating medium fermentation, and W342C mutant has a normal melibiose fermentation.

### Single-Cys mutations at the proposed cation-binding site of MelB_St_

**Figure 4:**
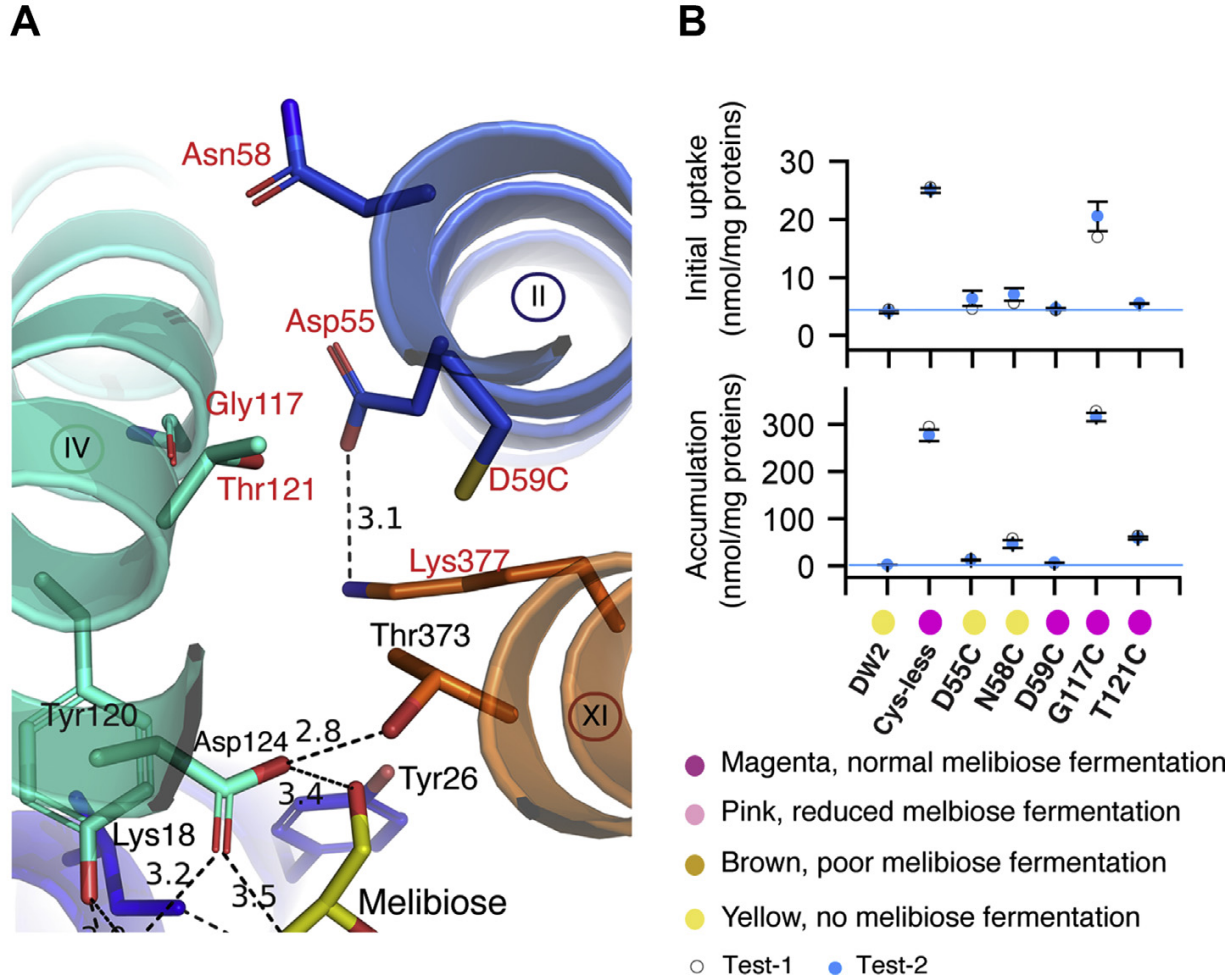
**Mutants in the proposed cation-binding site.***A*, Cys mutants in the proposed cation-binding site [PDB ID, 7L16]. Helices are shown in a *cartoon* representation and labeled in *Roman numerals*; positions important for cation binding are shown in *sticks*, labeled in three letters colored in *red*. H-bond and salt-bridge interactions are judged by distance that is <3.5 Å and indicated by *dashed lines*. Due to the limited structure resolution, the distance measurement was just used to estimate the relative proximity. *B*, melibiose transport and fermentation. [^3^H]Melibiose transport activity and melibiose fermentation results were from Figure 2. *Top*, initial uptake; *bottom*, the 5-min accumulation. *Magenta circle*, high melibiose fermentation; *yellow*, no melibiose fermentation.

While the cation binding in MelB was not structurally resolved yet, positions essential for the binding of Na^+^, Li^+^, and H^+^ have been functionally characterized, and all the three cations have a common binding pocket (34, 35, 36, 37) (Fig. 4*A*). Both Asp55 and Asp59 have been determined to be the ligands for Na^+^, and Asp59 is also the H^+^-binding residue (19, 25, 26, 27). Ala at Asn58 in a MelB homologue selectively eliminates the Na^+^-coupled transport with intact H^+^-coupled and Li^+^-coupled symport activities (38). D55C and D59C MelB_St_ mutants showed undetectable Na^+^-coupled melibiose active transport (Fig. 4*B*); however, the D59C mutant ferments melibiose at a normal rate as a uniport mutant that can bind and transport melibiose independent of cation (7). The N58C and T121C mutants also exhibited poor initial uptake, but the 5-min accumulation was 18% or 22% of the control. The single-Cys G117C mutant showed a greater melibiose accumulation than the control; notably, the G117C mutant at a WT background exhibits a twofold elevated melibiose transport *V*_max_ and 20-fold decreased Na^+^-binding affinity (39).

### Mapping 59 mutants that lost Na^+^/melibiose symport and melibiose fermentation

There are a total number of 62 mutants with no or poor melibiose fermentation grown as yellow and brown colonies, respectively. Among them, 59 mutants showed <15% initial uptake, <15% accumulation; three mutants including I22C, N58C, and L340C exhibited slightly higher accumulation than the Cys-less mutant (Table 3). Except for six mutants in the sugar-binding site and three in the cation-binding site, the remaining 50 mutants are nearly equally distributed in both helical bundles, more on helices I, V, VII, and X, which are discussed in the following sections.

**Table 3:** Distribution of 59 mutants^a^

59 single-Cys MelB_St_			Sugar site (6)	Cation site (3)	Other regions (50)
N-term bundle (34)	TMS	I (10)	K18, D19, I22		Y11, G12, G14, G17, T34, Y32, D35
		II (3)		D55, N58	G63
		III (4)			P78, G83, F95, H98
		IV (5)	D124, W128		W116, F127, L130
		V (7)	S153		R141, E142, L144, P148, A155, G171
		VI (3)			G178, F179, F190
	Periplasmic loops (1)				G172
	Cytoplasmic loops (1)				G74
Cytoplasmic middle-loop (0)					
C-Term bundle (25)	TMS	VII (5)			Y243, A246, Y256, Y257, Y260
		VIII (3)			L267, Y274, G276
		IX (1)			R295
		X (6)			G331, G337, F341, D351, D354, Y355
		XI (3)		K377	G379, A383
		XII (2)			P418, Y429
	Periplasmic loops (2)				Y396, P398
	Cytoplasmic loops (3)				N230, R363, Y433
C-Terminal tail (0)					

a that exhibit <15% initial uptake and <15% level of accumulation in the Na^+^-coupled melibiose transport activity, as well as no or poor melibiose fermentation. I22C, N58C, and L340C with slightly higher accumulation >15% of the Cys-less control.

#### Effects on the cytoplasmic salt-bridge network

**Figure 5:**
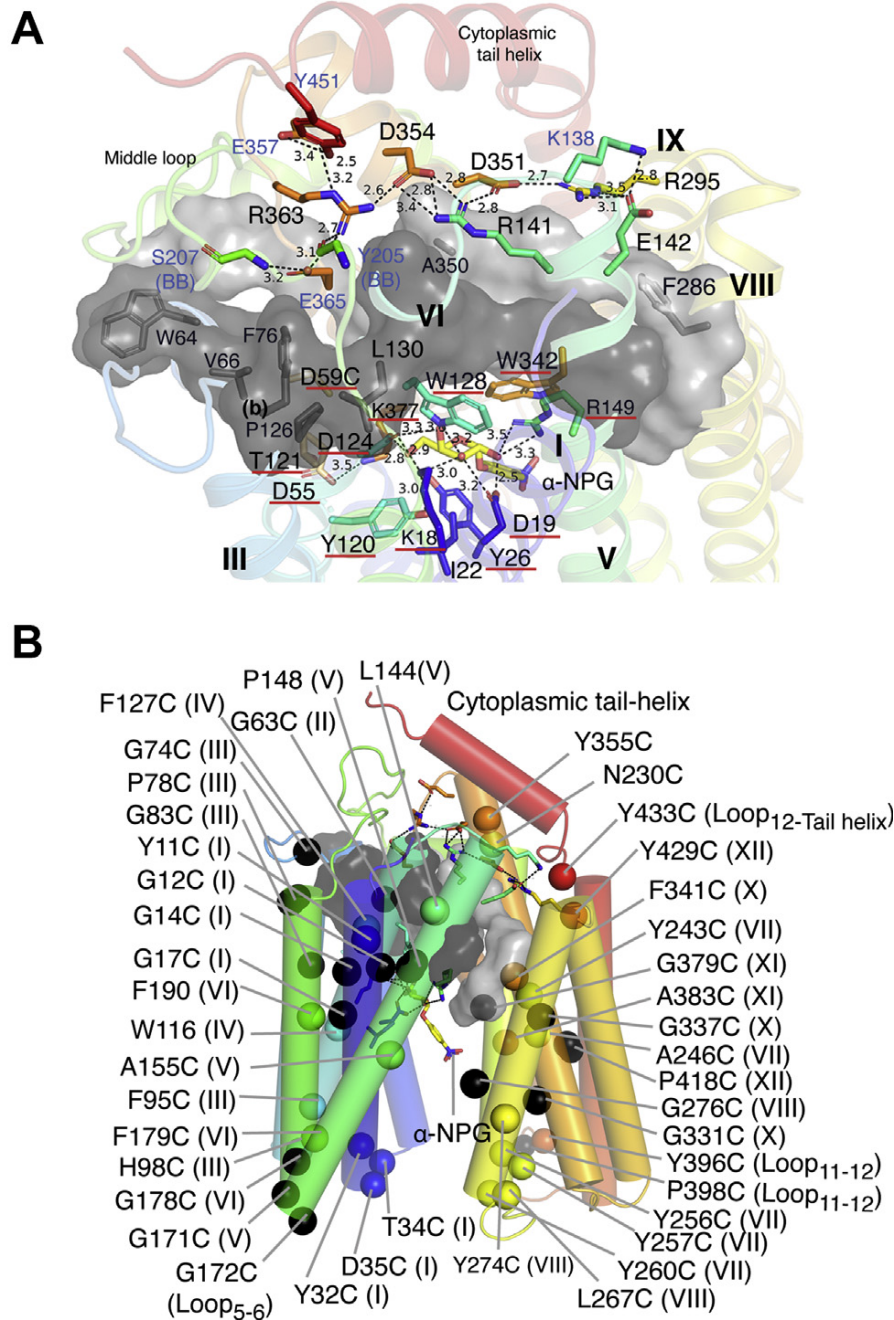
**Fifty mutants located outside the substrate binding sites [PDB ID,****7L17****].** Viewed from the cytoplasmic side, the salt-bridge network and ligand binding pocket are separated by an interbundle hydrophobic patch. Visible helices, middle loop, and C-terminal tail helix are labeled; side chains are shown in *sticks*; text labels for residues in the sugar- and cation-binding sites are highlighted with *red underline*; the bound α-NPG is shown in *yellow*. *A*, Cys mutants in the cytoplasmic salt-bridge network and hydrophobic patch. Nine charged residues [in loop_4–5_ (K138), helix V (R141 and E142), helix IX (R295), helix X (D351, D354, and E357), loop_10–11_(R363), and helix XI (E365), as well as three polar residues loop6–7 (Tyr205, Ser207) and C-terminal tail (Tyr451)] form a buried salt-bridge/hydrogen bond network at the cytoplasmic side of MelB_St_. The interbundle hydrophobic patch is shown in a surface representation from 11 residues in *dark gray* from the N-terminal bundle [helix II (W64, V66, and 8), helix III (F76), helix IV (I125, P126, W128, L130, and V131), and helix V (V145 and P146)] and ten residues in *light gray* from the C-terminal bundle [middle-loop helix (L220 and I223), helix VIII (L283 and F286), helix X (V346, I347, V349, and A350), and helix XI (I367 and Y369)]. *B*, mapping of 43 mutants that lost Na^+^/melibiose symport and melibiose fermentation on the crystal structure of MelB_St_. Mutant positions are indicated by Cα, and helix is indicated in *Roman numerals*.

In the 50-mutant list, there are seven on charged positions including one (Asp35) on the periplasmic end of helix I and six at the cytoplasmic side—Arg141 and Glu142 (helix V), Arg295 (helix IX), Asp351 and Asp354 (helix X), and Arg363 (the connecting loop_10–11_ between helices X and XI). The structure shows that those six cytoplasmic side-located charged residues dictate a wide-spread, buried, salt-bridge-dominated electrostatic network (Fig. 5*A*); notably, Arg141 (helix V) forms four salt-bridge interactions with Asp351 and Asp354 (helix X). These core interactions also recruit other three charged residues (Lys138, Glu357, and Glu365) forming a strong salt-bridge network on the cytoplasmic side of helices V, IX, X, XI, Loop_4–5_, and loop_10–11_. Mutants K138C, E357C, and E365C partially lost transport activities (Fig. 5*A* and Table S1).

#### Effects on interbundle hydrophobic patch

The structure showed that there are interbundle hydrophobic interactions that separate this cytoplasmic salt-bridge network from the sugar-binding pocket, as shown in surface representation colored in dark gray (N-terminal bundle) and light gray (C-terminal bundle), respectively (Fig. 5*A*). Twenty residues that involved in this hydrophobic patch are from the cytoplasmic ends of seven helices (II, III, IV, V, VIII, X, XI) and their connecting loops, including the cytoplasmic middle-loop helix. Out of the 20 positions, one is included in the 50-mutant list and three in the 105-mutant list.

Except for six positions in the cytoplasmic charge network and one in the interbundle hydrophobic patch, the majority of remaining 43 mutants contain a large number of Gly (13), Tyr (11), Phe (5), and Pro (4) (Table 3), and their locations are mapped onto the crystal structure (Fig. 5*B*). Notably, two Gly and one Pro residue are located in short helix-connecting loops or ß-turn structures: Gly74 on the cytoplasmic loop_2–3_, Gly172 on the periplasmic loop_5–6_, and Pro398 on the periplasmic loop_11–12_. For aromatic and other hydrophobic residues in the remaining 43-list, most are in the hydrophobic cores of each helical bundle or at ends of transmembrane helices (Fig. 5*B*).

#### Sensitive positions in the extramembrane regions

**Figure 6:**
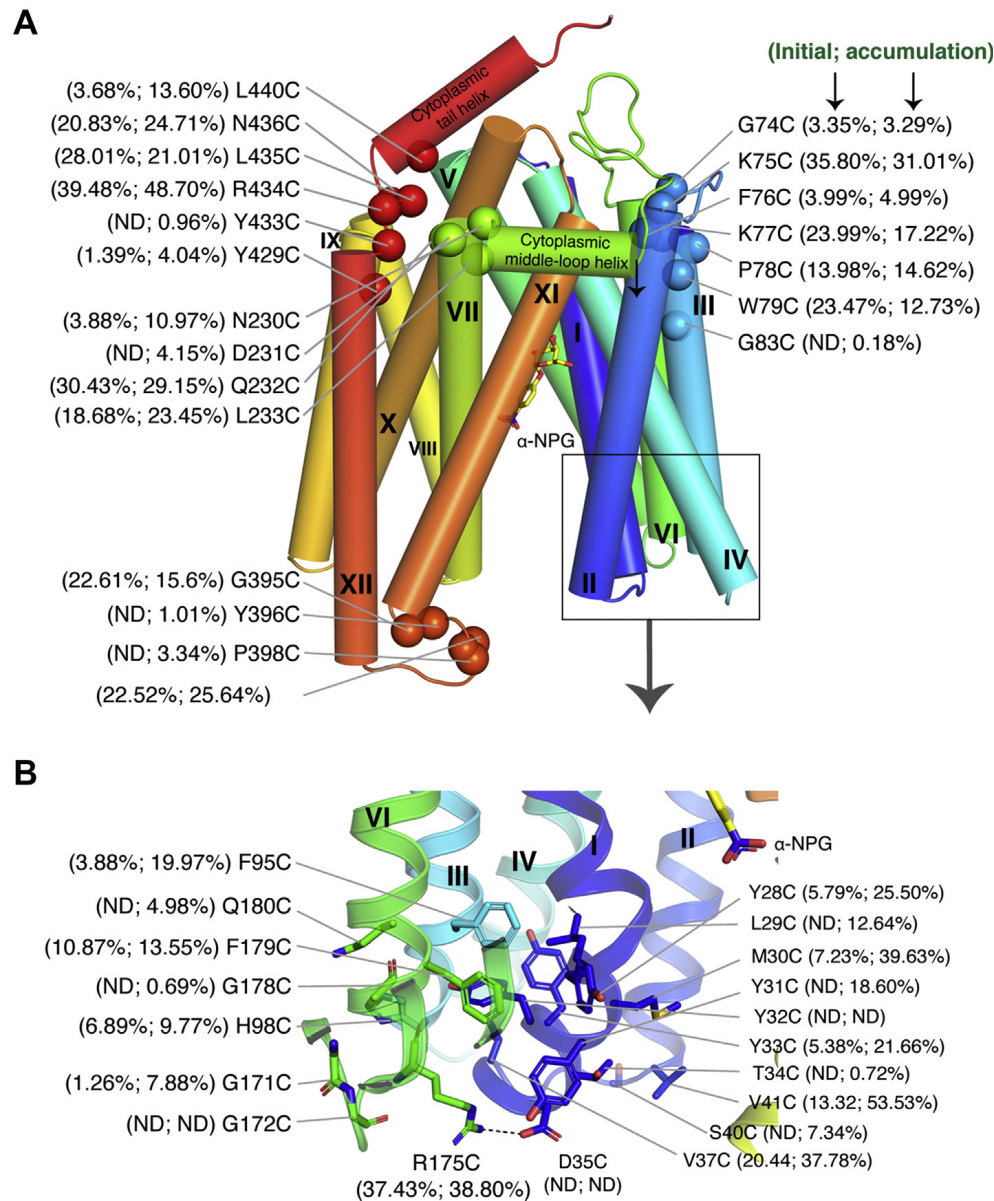
**Extramembrane regions important for melibiose transport.***A*, four areas at extramembrane loops or short turns between transmembrane helices or between cytoplasmic helices and transmembrane helices were mapped with single-Cys mutants with compromised melibiose transport activities as highlighted in ball on Cα position. *B*, the enlarged area from the *boxed region* as indicated by the *arrow*. Related initial uptake and 5-min accumulation of Na^+^-coupled melibiose transport were presented for each of the highlighted mutants based on the data shown in Figure 2. ND, no detectable melibiose transport. α-NPG is shown in *yellow*.

Surprisingly, many extramembrane regions do not tolerate single-Cys mutation well; the affected positions at extramembrane regions are often clustered together. At the cytoplasmic side, except for the mentioned cytoplasmic salt-bridge network around the extracellular regions of helix IV and X and the Loop_10–11_, there are other three hot spots. (i) The area around Asn230 forms a sharp turn between the cytoplasmic middle-loop helix and the transmembrane helix VII (Fig. 6). Single-Cys mutants at this stretch of positions 230 to 233 largely lost melibiose active transport with rates <25% of the control. Interestingly, D231C mutant does not catalyze active transport but still has a high melibiose fermentation (Fig. 2). (ii) The five-residue-long linker at positions 433 to 437 between the cytoplasmic tail-helix and the transmembrane helix XII. This region also contains several affected mutants. Y433C mutant transports melibiose poorly, R434C mutant exhibits medium activity, and all other four mutants have less than 30% of the control. Spatially, the two discussed stretches are in close proximity to the functional important cytoplasmic salt-bridge network. (iii) A stretch of residues spanning the loop_2–3_ (position 74–77) and other three positions (Phe76, Pro78, and Gly83) at the cytoplasmic side of helix II. Those regions are also sensitive to melibiose transport. The three F76C, P78C, and G83C mutants transport melibiose poorly, which are also in the 105-list (Fig. 6*A*).

On the periplasmic side, at least four out of six extracellular loops are important for transport or protein expression. The periplasmic end of the N-terminal bundle contains 18 mutants with poor activity, mapped on helices I, II, IV, and all three periplasmic loops (Loop_1–2_, III, IV, Loop_3–4_, and Loop_5–6_) (Fig. 6*B*). Half of these positions exhibit poor protein expression to varied levels, including Y32C (I), S40C (loop_1–2_), H98C (III), Gl72C (loop_5–6_). At the C-terminal side, only a stretch of positions on the loop_11–12_ contains two mutants (Y396C and P398C) that transport melibiose poorly and two less active mutants (G395C and N399C) at <25% of the control.

## Discussion

Cys-scanning mutagenesis has been successfully applied for the studies of structure and function of the H^+^-coupled lactose permease LacY (28, 40). With MelB of *E. coli*, few regions including helices I, VI, and XI, as well as Loop_10–11_, were also subjected to this technique (41, 42, 43, 44). Prior to an available structure, interpretations to these results at molecular levels were challenging, yet these Cys-scanning mutants in combination with site-directed mutagenesis and other biochemical/biophysical techniques offered the opportunities for identification of key residues that play essential roles in transport mechanisms (40, 45). These Cys-mutants were also served as a useful tool for the application of chemical modification with thiol-reactive agents for probing helical packing, cross-linking, and protein conformational dynamics (32, 40). In LacY, among the 401 unique single-Cys mutants (positions 2–402) covering 96% positions of the entire length of the protein, there are only 18 mutants that are unable to catalyze lactose accumulation as judged by <10% of Cys-less and an additional ten mutants that transport lactose <20% of Cys-less LacY (40). In MelB_St_, the data presented here showed that the number of positions that are sensitive to single-Cys mutations is surprisingly significantly greater than that observed in LacY. Among 475 unique single-Cys MelB_St_ mutants, there are 105 mutants at a transport initial rate <15% of Cys-less MelB_St,_ and among them, 59 mutants also failed in melibiose fermentation. The majority of affected positions are distributed throughout the protein with much less frequency on the middle loop, helix IX, and C-terminal tail. Apparently more sensitive positions on helix I are not surprising, because this helix carries four residues (Lys18, Asp19, Ile22, and Phe26) on the galactoside-binding pocket. Helix I also contains Tyr32, which is critical for protein expression, and Asp35, which is a suppressor site as discussed below. The protein sequence for the first two helices in MelB_St_ and MelB_Ec_ is nearly identical. The single-Cys mutations on positions Gly17, Lys18, Asp19, Tyr32, Thr34, or Asp35 of MelB_Ec_ also lost transport activity (41), highlighted the importance of helix I in MelB.

MelB_St_ is well expressed, and purified protein in solution is stable with a melting temperature (*T*_m_) of 65 °C (7, 20, 21). In this complete single-Cys library, a majority of mutants expressed MelB_St_ equivalent to the WT, but a few mutants showed poor expression. In particular, Y32C and G172C expressed the mutant protein at a level of 15% lower than that of the control. While more studies are needed to explain the expression defect, structurally Tyr32 on helix I forms three H-bonds with the backbone atoms of His98, Ala97, and Phe95 (helix III) and also forms multiple aromatic stacking interactions with Phe95 (helix III) and Phe179 (helix VI), as well as hydrophobic interactions with Val36 and Val37 on helix I (Fig. S2*A*). Thus, Tyr32, from the structural point of view, plays a critical role in stabilizing helical packing among helices I, III, and VI at their periplasmic end. The Y32C mutation could destabilize all these polar and hydrophobic interactions, which can affect MelB folding and protein stability. H98C mutant in this region was also poorly expressed (Fig. S2*A*). Gly172 is another position with affected protein expression and its adjacent G171C mutant also has poor transport activity with largely reduced protein expression. Both positions are not far from Tyr32 (Fig. S2*A*). The Gly172 is within a four-residue short loop called ß-turn that connects helices V and VI at the periplasmic side. ß-turn is a type of regular secondary structure element, which prefers Pro and Gly and has been shown to be important in protein stability (46). Cys replacement at Gly172 may interrupt the ß-turn structure, the helical packing, and then affect the protein stability. These results could suggest that the periplasmic end of N-terminal bundle in MelB_St_ plays an important role in the protein fold and/or stability.

Interestingly, the residues most frequently affected by Cys mutation are Tyr (68%) and Asp (44.44%). Tyr residues at a frequency of 80% prefer the transmembrane positions, while Asp residues only less than half (44.44%) in membrane domain. When analyzing the transmembrane-located positions, the affected frequencies for both Tyr and Asp are as high as 80% and 100%, respectively; *i.e.*, none of the transmembrane-located Asp residues tolerates Cys replacement. However, Cys replacements on more than half of all other 14 charged residues (Arg, Lys, and Glu) in transmembrane domain (14/21) still maintain melibiose transport to varying degrees. These 14 charged residues are located at the end of helix or the out-membrane extension of the longer helix (Fig. S2*B*) and likely play varied roles in MelB_St_ solubility.

The structure and functional analyses showed that the transmembrane-located Asp residues provide the most important side chains for MelB function. Among the eight transmembrane Asp residues, Asp19 (helix I) and Asp124 (helix IV) are the two most critical residues in the sugar-specificity determinant pocket. They accept multiple H-bonds from all four hydroxyl groups (C2-, C3-, C4-, and C6-OH groups) on the galactosyl moiety. Cys replacement at any position completely eliminated active transport and fermentation of melibiose (Figs. 2 and 3). Asp55 and Asp59 (helix II) are the two major ligands of cation Na^+^ or Li^+^, and Asp59 is likely the H^+^-binding site (19, 25, 26, 27). In addition to the four Asp residues in the N-terminal helical bundle, there is another functionally important Asp35 at the periplasmic end of helix I. Asp35 is also close to Tyr32, and Cys mutations at several adjacent positions can significantly reduce the expression of MelB_St_. Glu replacement on periplasmic-side Asp35 is a suppressor, which can greatly rescue transport activities of inactive mutants located on the opposite side of the protein. These primary mutation sites can be any of three Arg (Arg141, Arg295, and Arg363), and their mutations can be varied (47). The three Arg residues are engaged in the cytoplasmic salt-bridge/H-bonding network with Asp351 and Asp354 on helix X. It would be interesting to test if the D35E could also rescue melibiose transport of D351C, D354C, or other inactive mutants in this important area. In MelB_Ec_, these Asp residues have been also showed to be important for melibiose transport activities (26, 41, 43, 48).

Asp231 at the beginning of transmembrane helix VII is another Asp in this 105-list (Fig. 6). The single-Cys mutants at the adjacent positions Asn230 and Gln232 also had reduced activities of <20% of the Cys-less control. Asp231 forms interactions with helix X through multiple salt-bridge/H-bonding with Lys359 and Tyr355, and those two positions are located at the opposite face of Asp351 and Asp354 on helix X that are essential for the salt-bridge/H-bonding network (Fig. S2*C*). Notably, Tyr355 is also a poor-transport mutant. Asp231 also forms a salt-bridge with Arg434 on the connecting loop between helix XII and the C-terminal tail, as well as an intra-helix H-bond with Gln222. The adjacent Asn230 of this Asp231 forms a H-bond with the backbone of The352 (helix XI). These sensitive positions to Cys mutations form a cluster around the end of helix X and the short linker between the middle-loop helix and helix VII, indicating its importance in the transport mechanism. Since it is far from the cation or sugar binding sites, it is expected that this region is engaged in the conformational dynamics.

Tyr side chain carries an aromatic ring and a polar group, good for stronger helical packing as illustrated for Tyr32 (Fig. S2*A*). Other examples are also shown here with Tyr11 (Fig. S2*D*). Tyr11 on helix I interacts with helices IV and V by forming H-bonds with the backbone N atom of the sugar-binding residues Trp128 and backbone O atom of the Phe127 on helix IV, respectively; Tyr11 is also surrounded by a group of hydrophobic atoms (helix V) and aromatic residues (helix IV) including Phe127, Trp128, Leu144, and Pro148. Structurally, Tyr11 plays an important role in stabilizing the helical packing between I and IV and presenting Trp124 (helix IV) and Lys18 to donate H-bonds to galactosyl moiety. It is worth noting that all shown positions close to Tyr11 are in the list of 105 mutants with poor transport activities. It is possible that these loss-of-function Cys mutants, except at positions Tyr120, Trp128, Lys18, and Asp19, may destabilize the core structure of this helical bundle. It will be interesting to test the mutational effects of those positions on ligand binding. Overall, Asp and Tyr residues play important structural and functional roles in melibiose transport mediated by MelB_St_.

In conclusion, MelB_St_ function is sensitive to Cys mutagenesis, and the affected positions not only exist in the substrate specificity determinant pockets, but are also often found outside the binding sites. Furthermore, many loss-of-function mutation positions outside the substrate sites may directly or indirectly relate to the cytoplasmic salt-bridge/H-bonding network. It is likely that the protein stability and conformational changes of MelB_St_ required for transport are likely to involve a wide range of sites in both of the N- and C-terminal domains, which are in agreement with the notion that both N- and C-terminal domains move around the bound substrates as proposed previously (49, 50). This complete library of 476 unique single-Cys mutants will be a useful tool to explore the cation-coupled symport mechanism and conformational dynamics of MelB, as well as facilitate the studies on its human homologues, such as a lipid transporter MFSD2A in the brain and eyes.

## Experimental procedures

### Materials

[1-^3^H]Melibiose was custom synthesized by *PerkinElmer*. Melibiose was purchased from *Acros Organics* (*Fisher Scientific*). MacConkey agar media (lactose free) was purchased from Difco. All other materials were reagent grade and obtained from commercial sources. Oligodeoxynucleotides were synthesized by Integrated DNA Technologies.

### Bacterial strains and plasmids

*E. coli* DW2 strain (*melA*^+^, Δ*melB*, Δ*lacZY*), obtained from Dr Gérard Leblanc, was used for the transport assay or melibiose fermentation. *E. coli* XL1 Blue cells were used for plasmid preparation. The expression plasmid pK95 ΔAH/ClessMelB_St_/CHis_10_ encoding the Cys-less MelB_St_ with a His_10_-tag at the C-terminus (37, 51) was generated by placing Ala at four endogenous Cys positions (110, 235, 310, 363). This plasmid was used as the template for constructing the single-Cys mutant by placing a Cys to each position, one at a time, which yielded a complete library of 476 single-Cys MelB_St_ mutants. All mutants were constructed by *QuickChange Site-Directed Mutagenesis Kit* (*Invitrogen*) and confirmed by DNA sequencing analysis.

### Cell growth for transport assay and protein expression determination

*E. coli* DW2 cells containing a given plasmid were grown in Luria-Bertani (LB) broth with 100 mg/l of ampicillin in a 37 °C shaker overnight. The overnight cultures were diluted by 5% to LB broth supplemented with 0.5% glycerol and 100 mg/l of ampicillin, and constitutive overexpression was obtained by shaking at 30 °C for 5 h.

### [1-^3^H]Melibiose transport assay

*E. coli* DW2 cells expressing MelB_St_ were washed with 50 ml of 100 mM KP_i_ (pH 7.5) twice, followed by washing with assay buffer (50 ml 100 mM KP_i_, pH 7.5, 10 mM MgSO_4_). The cell pellets were resuspended with the assay buffer and adjusted to A_420_ = 10 (∼0.7 mg protein/ml). Fifty microliter of cells was mixed with 2 μl of 10 mM [^3^H]melibiose at a specific activity of 10 mCi/mmol and 500 mM NaCl (at a final concentration of 0.4 mM melibiose and 20 mM NaCl). Each transport dataset for all single-Cys mutants including the positive control Cys-less mutant and the negative control DW2 cells without MelB contains three time points at 0, 10 s, and 5 min, respectively. The transported melibiose was collected *via* dilution and fast filtration and counted using a liquid scintillation counter. Activity was converted to nmol melibiose/mg proteins or normalized to percent of Cys-less mutant after corrected by zero point and negative control.

### Melibiose fermentation

Each of the single-Cys plasmids was transformed into *E. coli* DW2 cells (Δ*melBΔlacYZ*), plated on MacConkey agar plates containing 30 mM melibiose and 100 mg/l of ampicillin, and incubated at 37 °C (39, 52). After 18 h, the plates were viewed and photographed immediately. Formation of red/pink colonies after 18 h is indicative of melibiose fermentation; brown/yellow colony phenotype indicates the cells’ inability to ferment melibiose.

### SDS-16%PAGE and western blotting

Intact cells grown as for the transport assay were washed with 20 mM Tris-HCl, pH 7.5; 10 μl of cells at A_600_ = 15 were diluted to 40 μl, then sonicated in an ice-cold water bath (Branson 2510) for 5 min 20 μl of the whole cell lysate (equivalent to 7 μg protein as judged by protein assay) was loaded onto SDS-16%PAGE. The gel was transferred onto PVDF membrane by the Trans-Blot Turbo transfer system (Bio-Rad Laboratories) at 1.3 A, 25 V for 20 min. The blocked PVDF membrane by 3% BSA was then reacted with the penta-His *antibody* (*Invitrogen*), and washed in 20 mM Tris-HCl, pH 7.5, and 500 mM NaCl for 30 min, and further probed by the secondary Alexa-fluor 680 antibody (*Invitrogen*) diluted in 3% BSA, 0.1% Tween-20, and 0.01% SDS for 1 h. After washing with 20 mM Tris-HCl, pH 7.5, and 500 mM NaCl for 30 min, the membrane was scanned by LI-COR Odyssey Infrared Image system at 700 nm. Molecular weight markers were indicated (*Precision plus protein WesternC standards*, Bio-Rad Laboratories).

## Data availability

The raw data for all figures and tables are available from the corresponding author on reasonable request.

## Supporting information

This article contains supporting information.

## Conflict of interest

The authors declare that they have no conflicts of interest with the contents of this article.
