# Supplementary material for: Complete cysteine-scanning mutagenesis of the Salmonella typhimurium melibiose permease
Source: J Biol Chem. 2021 Aug 18;297(3):101090. doi: 10.1016/j.jbc.2021.101090 (PMC8437787; doi:10.1016/j.jbc.2021.101090)
Supplement: Supplemental Figures S1, S2 and Table S1 [file mmc1.pdf]

Supporting Information

Complete cysteine-scanning mutagenesis of the *Salmonella typhimurium* melibiose permease

Kelsey J. Markham, Elena B. Tikhonova[#], Aaron C. Scarpa, Parameswaran Hariharan, Satoshi Katsube, and Lan Guan[§]

Department of Cell Physiology & Molecular Biophysics, Center for Membrane Protein Research, School of Medicine, Texas Tech University Health Sciences Center, School of Medicine, Lubbock, Texas, 79430

[§]To whom correspondence should be addressed: Lan Guan, Department of Cell Physiology & Molecular Biophysics, Texas Tech University Health Sciences Center, 3601 4th Street, STOP 6551, Lubbock, Texas 79430.

[#]Current address: Department of Cell Biology and Biochemistry, Texas Tech University Health Sciences Center, School of Medicine, Lubbock, Texas, 79430

Telephone: (806) 743-3102
Fax: (806) 743-1512
E-mail: **Lan.Guan@ttuhsc.edu**

Contents:

SI Table 1. Expression and transport activities.

SI Figure 1a-d. Melibiose transport and fermentation.

SI Figure 2. Mapping mutations.

SI Table 1. Expression and transport activities

Helix	Mutant	Expression (%)	Initial uptake (%)	SE	Accumulation (%)	SE	Melibiose fermentation
	DW2		0.06	0.06	0.07	0.07	● yellow
	C-Less	100.00	100.00	6.17	100.00	5.19	● magenta
Helix I	Y11C	61.20	1.06	3.24	1.38	0.14	● yellow
	G12C	76.36	1.54	0.14	0.53	0.30	● yellow
	G14C	67.36	4.79	2.98	3.45	0.27	● yellow
	A15C	53.43	2.66	3.37	27.48	0.98	● magenta
	G17C	69.62	-10.38	6.31	0.19	0.26	● yellow
	K18C	56.35	-21.35	0.08	-0.93	0.13	● ochre
	D19C	68.20	-23.63	0.54	-0.89	0.06	● yellow
	I22C	106.22	-20.36	2.58	22.54	0.36	● ochre
	I24C		14.02	3.90	13.11	0.10	● magenta
	V25C	81.54	4.19	1.68	7.73	0.95	● magenta
	Y26C	89.38	-22.49	2.45	0.80	0.05	● magenta
	Y28C	90.28	5.79	0.84	25.50	0.11	● pink
	L29C	86.79	-8.37	4.32	12.64	0.35	● magenta
	M30C	86.20	7.23	0.11	39.63	0.47	● magenta
	Y31C	97.37	-1.45	1.79	18.60	0.84	● magenta
	Y32C	14.03	-27.64	1.00	-1.11	0.00	● yellow
	Y33C	71.65	5.38	0.74	21.66	0.46	● magenta
	T34C	69.47	-24.30	2.21	0.72	0.08	● yellow
	D35C	69.47	-24.23	0.21	-0.96	0.10	● yellow
Peri-loop	L39C	88.37	10.57	0.32	38.18	0.05	● magenta
Helix II	S40C	31.62	-17.47	2.35	7.34	0.45	● magenta
	V41C	89.81	13.32	13.38	53.53	0.31	● magenta
	R52C	92.61	-6.22	1.27	0.18	0.17	● magenta
	D55C	67.66	2.72	4.92	3.39	0.22	● yellow
	N58C	101.49	7.70	4.03	18.07	2.32	● yellow
	D59C	100.17	-3.07	0.24	0.93	0.03	● magenta
	G63C	99.71	0.57	1.04	1.46	0.07	● yellow
	V66C		11.31	2.29	11.26	0.39	● magenta
	G74C	63.25	3.35	0.24	3.29	0.37	● yellow
Helix III	F76C	89.70	3.99	2.13	4.99	0.04	● pink
	P78C	79.85	13.98	0.08	14.62	0.17	● yellow
	G83C	56.67	-0.95	0.37	0.18	0.16	● yellow
	N87C	67.37	12.05	1.91	17.31	2.03	● magenta
	F95C	49.91	-0.10	1.85	2.43	0.01	● yellow
	H98C	35.00	6.89	1.04	9.77	0.21	● yellow
Helix IV	Y113C	84.29	-5.56	10.59	10.94	0.29	● magenta
	W116C	67.69	-13.20	0.94	2.69	0.07	● yellow
	Y120C	87.87	-18.10	1.53	-0.28	0.14	● pink
	T121C	77.75	3.10	0.46	21.48	0.82	● magenta
	D124C	83.97	-20.62	0.41	-0.82	0.01	● yellow
	P126C	85.98	6.82	4.09	32.91	0.72	● magenta
	F127C	49.15	-12.29	0.09	0.03	0.04	● yellow
	W128C	105.78	-18.00	0.33	-0.78	0.02	● yellow
	L130C	89.06	0.60	1.70	9.37	0.04	● yellow
	I134C	97.91	3.09	2.44	5.97	0.46	● pink
	T135C	95.66	1.89	2.44	5.89	0.46	● magenta
	L136C	82.46	6.89	1.09	9.69	0.33	● magenta
Helix V	R141C	81.20	-2.88	1.03	0.44	0.04	● yellow
	E142C	88.51	-2.33	0.81	1.79	0.17	● yellow
	L144C	97.14	-7.44	0.90	6.92	0.00	● yellow
	P148C	116.05	1.17	2.26	4.04	0.04	● yellow
	R149C	93.29	-6.83	0.26	0.86	0.09	● pink
	S153C	90.93	-14.49	0.77	0.17	0.05	● yellow
	A155C	94.34	-2.21	0.00	3.10	0.12	● yellow
	G171C	57.67	1.26	10.98	7.88	1.03	● yellow
	G172C	14.88	-17.59	1.73	-0.62	0.21	● yellow
Helix VI	G178C	50.44	-7.59	0.68	0.69	0.18	● yellow
	F179C	57.16	10.87	0.18	13.55	4.99	● yellow
	Q180C	43.05	-3.34	0.50	4.98	0.47	● pink
	F190C	90.13	1.07	0.30	11.99	0.51	● yellow
	N230C	76.18	3.88	9.31	10.97	0.14	● yellow
Helix VII	D231C	69.56	-2.12	5.36	4.20	0.01	● magenta
	L241C	61.50	-1.65	2.04	3.53	0.83	● pink
	Y243C	48.99	-8.81	1.85	0.19	0.11	● yellow
	N244C	103.92	5.55	1.54	6.69	0.04	● magenta
	A246C	67.74	-7.50	2.53	-0.15	0.16	● ochre
	N251C	80.65	0.63	5.68	11.65	0.33	● magenta

	Y256C	63.29	-3.46	1.64	1.85	0.00	● (yellow)
	Y257C	43.39	-10.04	0.34	0.68	0.71	● (yellow)
	F258C	77.73	14.28	7.23	8.50	0.73	● (magenta)
	Y260C	66.51	-0.06	0.53	4.71	0.18	● (yellow)
Helix VIII	L267C	77.80	1.35	2.27	3.05	0.01	● (brown)
	Y274C	100.46	2.36	1.86	10.52	0.12	● (brown)
	G276C	85.48	-2.68	4.10	1.37	0.46	● (yellow)
	A278C	74.50	13.82	1.26	18.55	1.15	● (magenta)
	F286C	93.59	1.41	1.56	0.65	0.02	● (yellow)
Helix IX	R295C	89.59	-14.69	1.56	-0.42	0.02	● (yellow)
	N323C	103.75	3.73	1.65	25.13	0.56	● (magenta)
Helix X	G331C	34.14	-13.67	0.23	-0.34	0.01	● (yellow)
	G337C	12.60	-8.15	1.69	-0.49	0.10	● (yellow)
	A339C	103.16	-1.60	1.06	3.59	0.11	● (magenta)
	L340C	88.26	6.88	0.32	16.71	0.20	● (yellow)
	F341C	72.69	0.30	2.11	3.39	0.77	● (yellow)
	W342C	112.41	-5.61	1.40	2.60	0.13	● (magenta)
	V343C	103.25	4.64	0.98	12.28	0.77	● (magenta)
	L344C	93.17	9.51	0.42	12.10	0.19	● (magenta)
	A350C	99.72	2.88	4.46	9.80	0.05	● (yellow)
	D351C	69.42	-2.85	5.37	0.48	0.07	● (yellow)
	D354C	77.47	5.50	1.23	3.14	0.29	● (yellow)
	Y355C	77.26	0.78	3.79	1.26	0.08	● (yellow)
Cyto-loop	R363C	86.77	0.81	3.79	0.26	0.08	● (yellow)
Helix XI	S366C	123.72	0.42	2.24	4.71	0.90	● (magenta)
	Q372C	110.22	3.86	0.32	1.48	0.20	● (pink)
	K377C	54.30	-9.28	2.59	0.22	0.14	● (yellow)
	G378C	37.44	2.70	2.58	1.25	0.02	● (magenta)
	G379C	92.86	-3.49	2.01	-0.22	0.06	● (yellow)
	A381C	117.21	13.57	14.82	27.05	0.21	● (magenta)
	A383C	74.26	11.66	12.02	14.79	2.04	● (yellow)
Peri-Loop	Y396C	75.36	-2.34	2.77	1.01	0.11	● (yellow)
Peri-Loop	P398C	69.54	-14.08	0.62	3.34	0.06	● (yellow)
Helix XII	P418C	69.88	-15.32	0.32	0.34	0.06	● (yellow)
	F422C	16.80	-6.33	0.51	0.16	0.39	● (magenta)
	Y429C	82.89	1.39	5.07	4.04	0.12	● (yellow)
	Y433C	71.12	-7.31	3.09	0.96	0.00	● (yellow)
Cyto-Tail	L440C	76.11	3.68	0.61	13.60	0.65	● (pink)

Protein expression was expressed as a percentage of the internal control Cys-less $MelB_{St}$ mutant included in each western blots. Initial uptake and accumulation were measured at 10 sec and 5 min, respectively.

- ● Magenta, high melibiose fermentation
- ● Pink, reduced melbiose fermentation
- ● Brown, poor melibiose fermentation
- ● Yellow, no melibiose fermentation

SI Figure 1a. Melibiose transport and fermentation (Position 2-100)

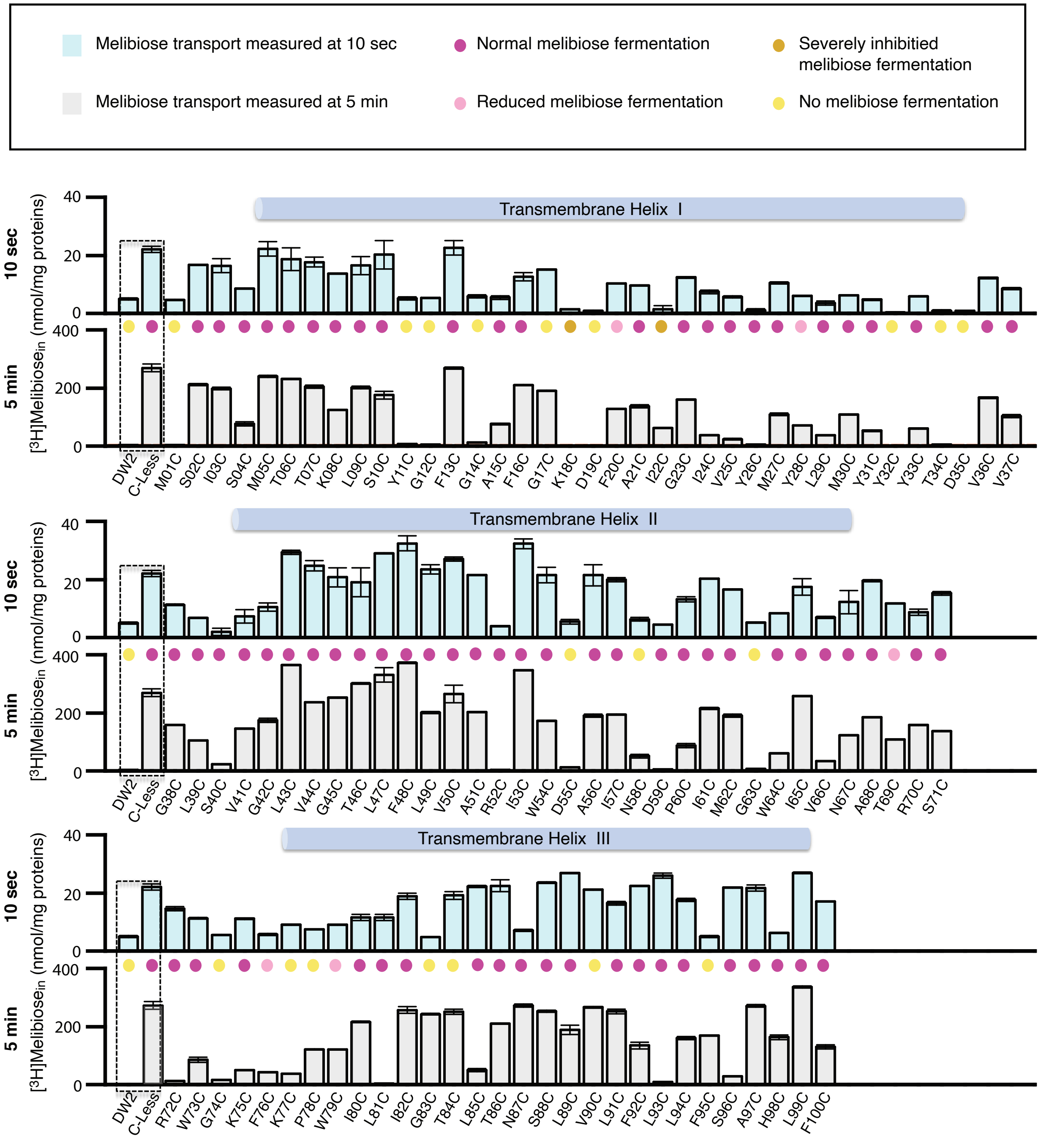

SI Figure SI 1b. Melibiose transport and fermentation (Position 101 - 229)

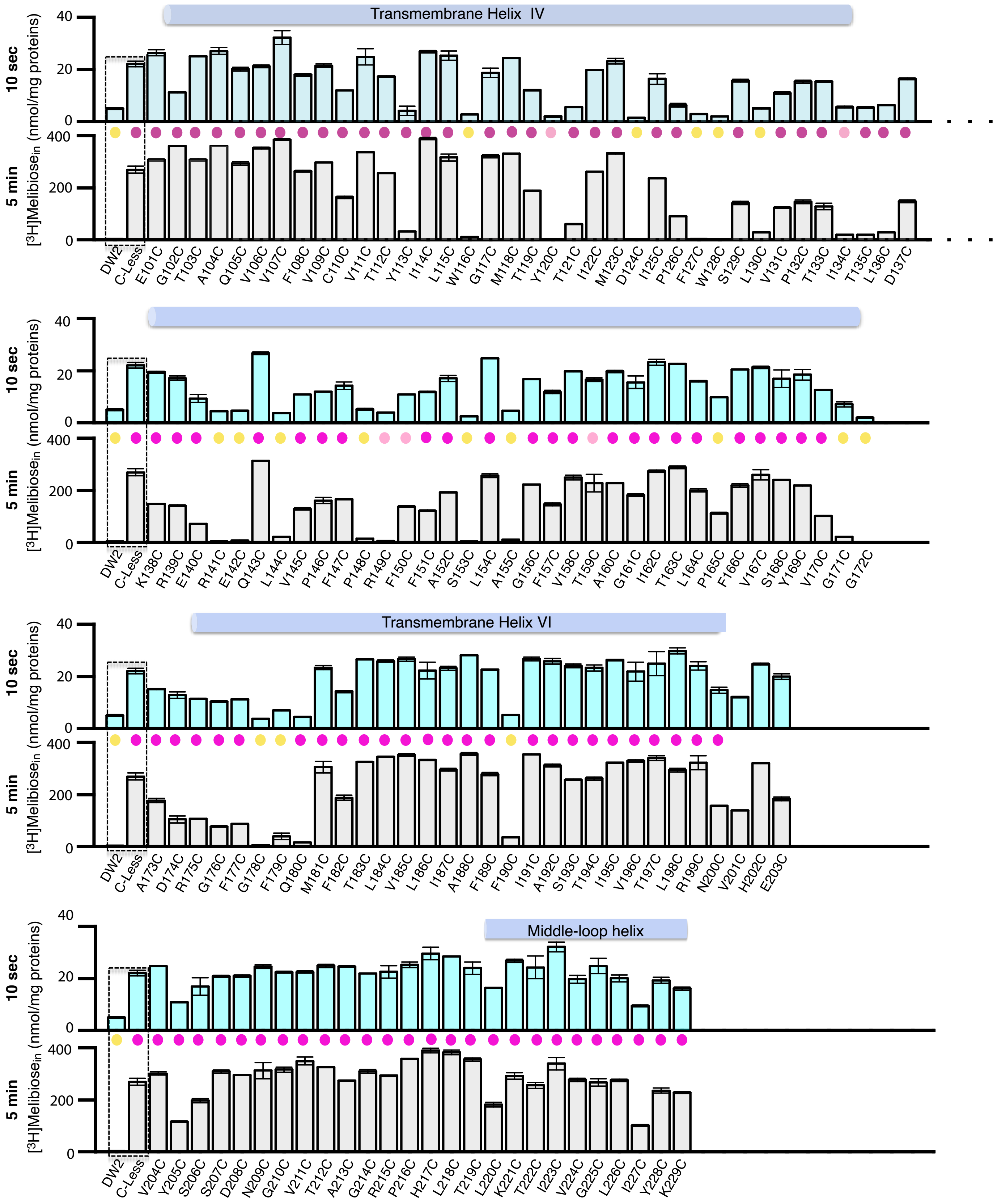

SI Figure 1c. Melibiose transport and fermentation (Position 230 - 363)

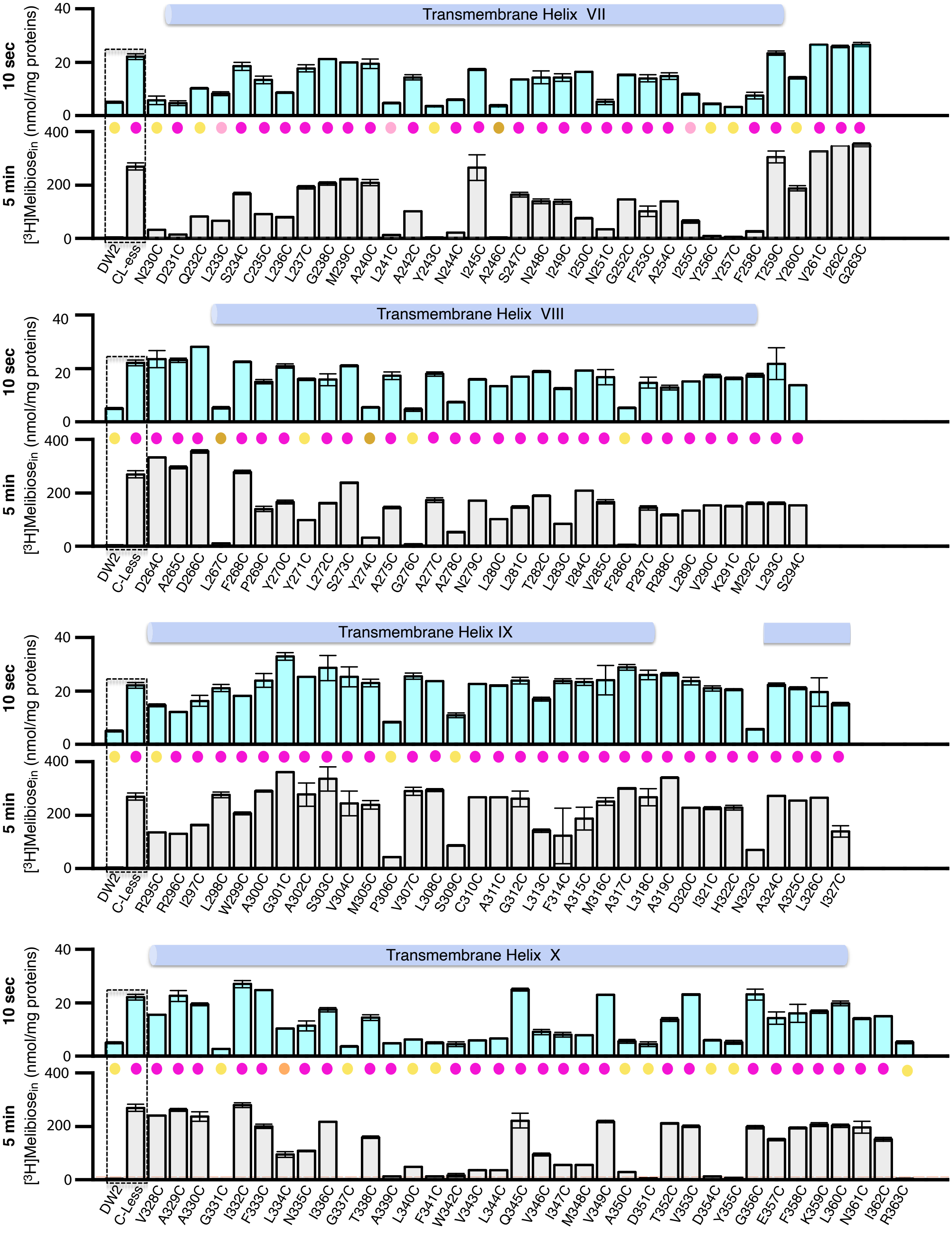

SI Figure 1d. Melibiose transport and fermentation (Position 364 - 476)

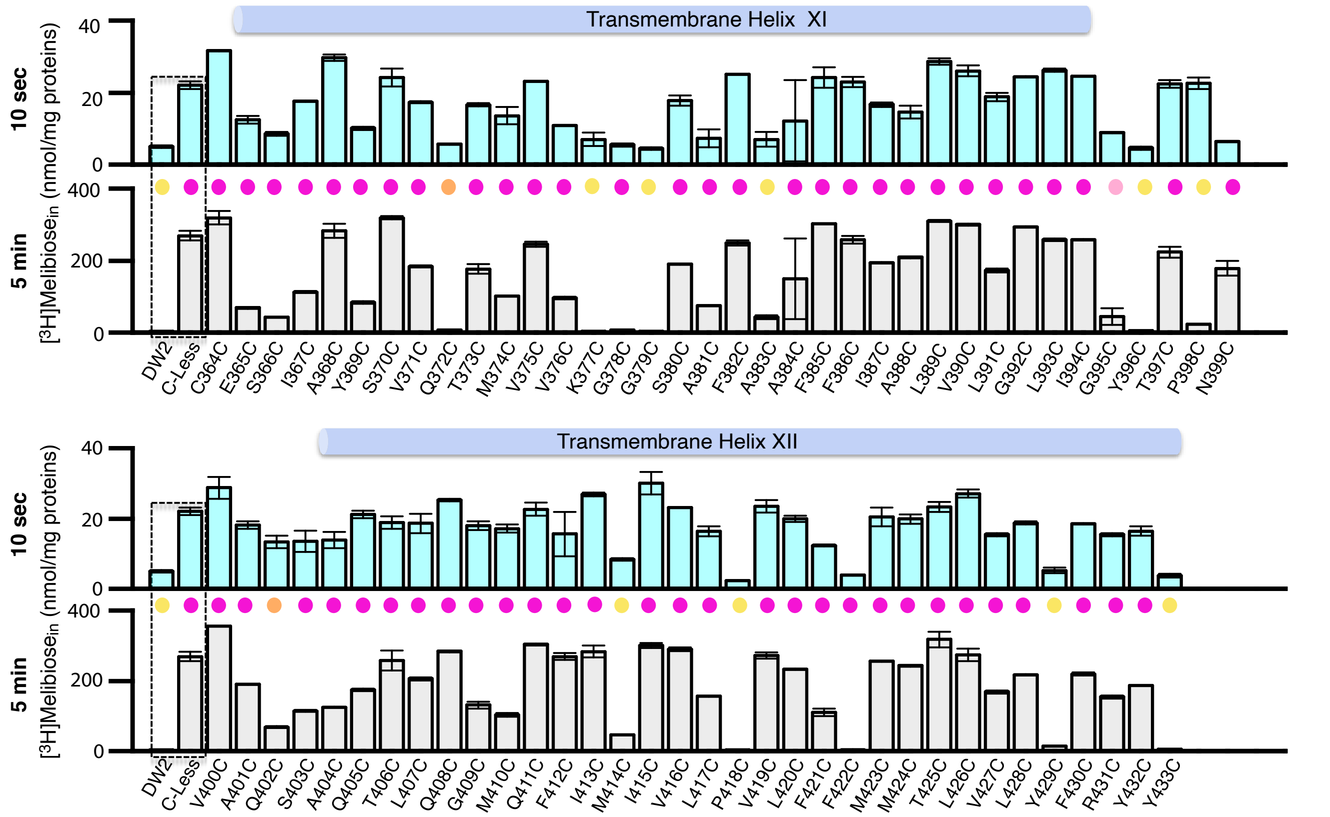

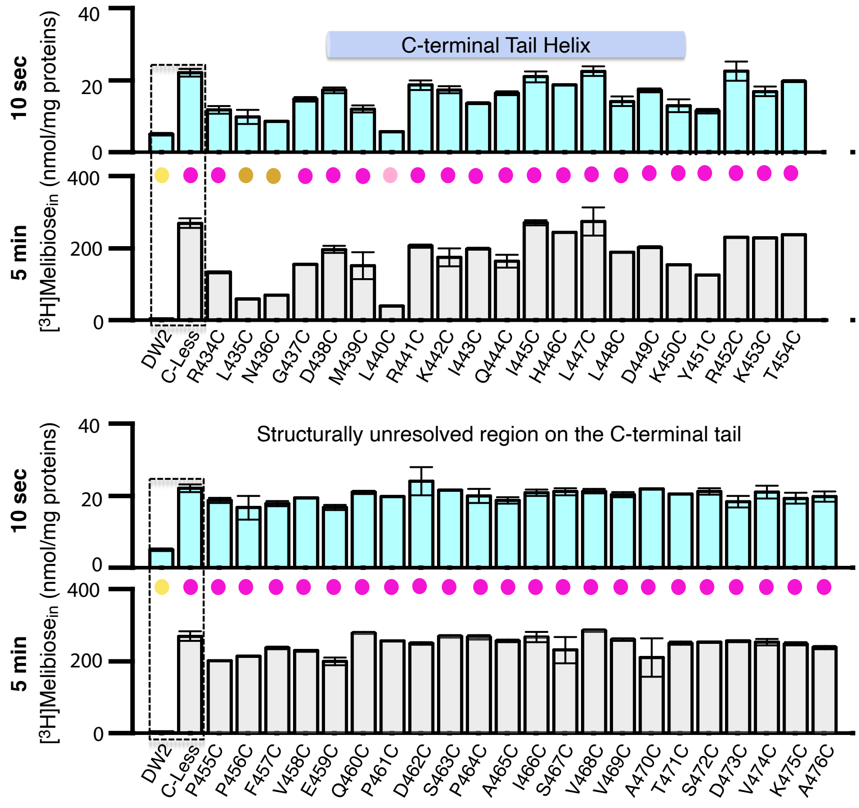

SI Figure 1. Melibiose transport and fermentation. Melibiose transport initial uptake at 10 sec (top panels) and 5-min accumulation (lower panels) were presented in histogram bars after subtracted from zero-point values. Melibiose fermentation results for each mutants were presented by four groups in color-filled circles as indicated on the first page. The full dataset is splitted into 4 pages covering 2-100, 101-229, 230-363, and 364-476. DW2, a *E. coli* strain (*melA*$^+$*B*$^-$*lacZ*$^-$*Y*$^-$) with no plasmid. C-Less, Cys-less MelB$_{St}$ mutant as the positive control. The position for helix is indicated according the crystal structure of D59C MelB$_{St}$ (PDB ID 7L17).

SI Figure 2

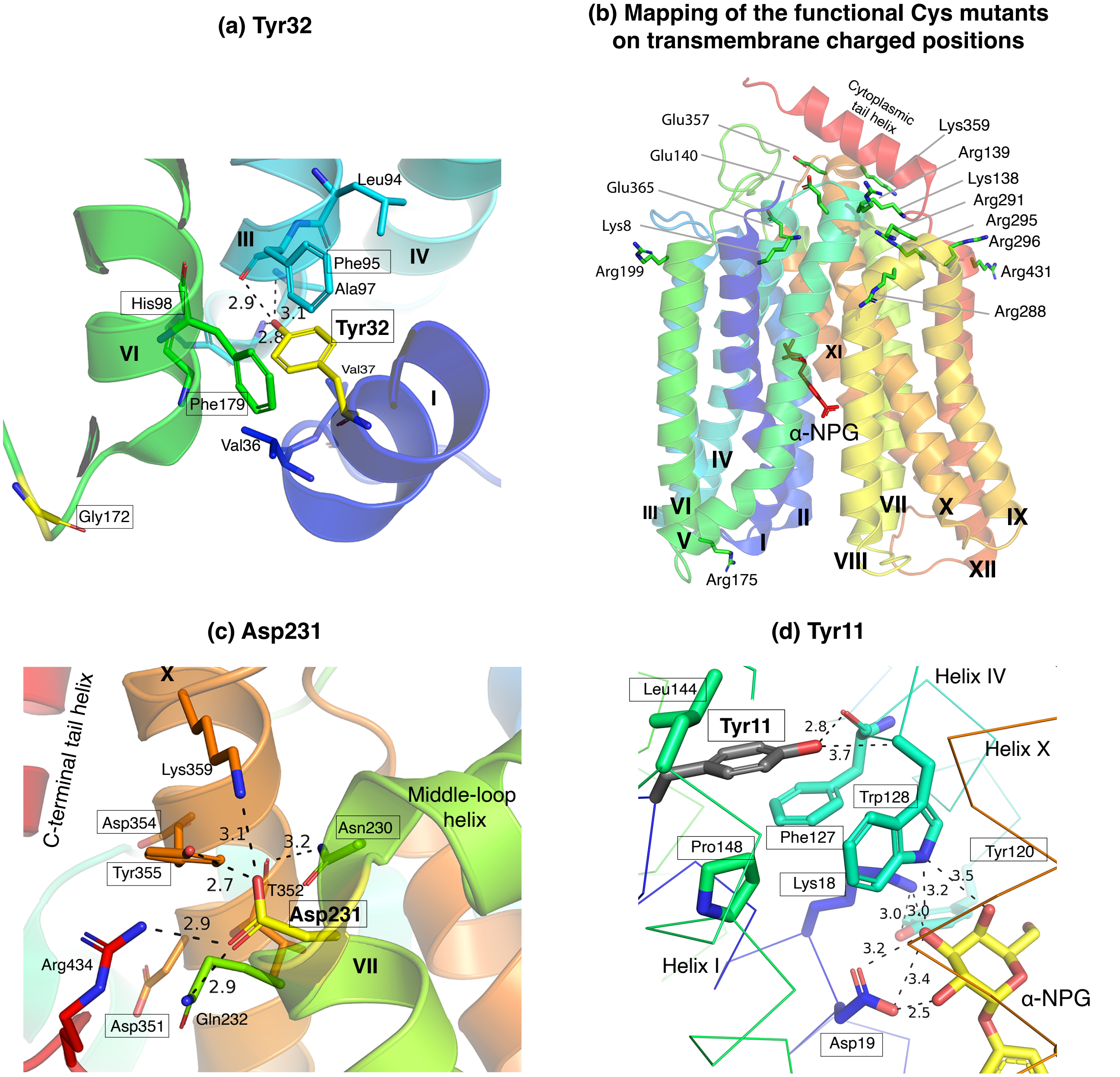

SI Figure 2. Mapping mutations. All panels were prepared from the D59C $MelB_{St}$ structure [PDB ID, 7L17]. Helices are labeled in Roman numerals. **(a) Tyr32.** The interactions surrounding Tyr32 at the periplasmic end of N-terminal bundle are highlighted with sticks. **(b) Mapping of the functional Cys mutants on transmembrane charged positions.** **(c) Asp231.** The interactions surrounding Asp231 at the cytoplasmic side of the C-terminal bundle are highlighted with sticks. **(d) Tyr11.** The interactions surrounding Tyr11 at the cytoplasmic side of the N-terminal bundle are highlighted with sticks. Broken lines, H-bond; box, mutants with initial uptake <15% of the Cys-less control in the 105 mutant list.
